# SARS-CoV-2 antibody prevalence among homeless people and shelter workers in Denmark: a nationwide cross-sectional study

**DOI:** 10.1186/s12889-022-13642-7

**Published:** 2022-06-27

**Authors:** Alexandra R Röthlin Eriksen, Kamille Fogh, Rasmus B. Hasselbalch, Henning Bundgaard, Susanne D. Nielsen, Charlotte S. Jørgensen, Bibi F. S. S. Scharff, Christian Erikstrup, Susanne G. Sækmose, Dorte K. Holm, Bitten Aagaard, Jonas H. Kristensen, Cecilie A. Bødker, Jakob B. Norsk, Pernille B. Nielsen, Lars Østergaard, Svend Ellermann-Eriksen, Berit Andersen, Henrik Nielsen, Isik S. Johansen, Lothar Wiese, Lone Simonsen, Thea K.Fischer, Fredrik Folke, Freddy Lippert, Sisse R. Ostrowski, Steen Ethelberg, Anders Koch, Anne-Marie Vangsted, Tyra Krause, Anders Fomsgaard, Claus Nielsen, Henrik Ullum, Robert Skov, Kasper Iversen

**Affiliations:** 1 Department of Cardiology, Copenhagen University Hospital, Herlev and Gentofte, Borgmester Ib Juuls Vej 1, 2730 Herlev, Denmark; 2 Department of Emergency Medicine, Copenhagen University Hospital, Herlev and Gentofte, Herlev, Denmark; 3 Department of Clinical Medicine, University of Copenhagen, Copenhagen, Denmark; 4 Department of Cardiology, Copenhagen University Hospital, Rigshospitalet, Copenhagen, Denmark; 5 Department of Infectious Diseases, Copenhagen University Hospital, Rigshospitalet, Copenhagen, Denmark; 6 Statens Serum Institut, Copenhagen, Denmark; 7 Department of Clinical Immunology, Copenhagen University Hospital, Rigshospitalet, Copenhagen, Denmark; 8 Department of Clinical Microbiology, Aarhus University Hospital, Aarhus, Denmark; 9 Department of Clinical Medicine, Aarhus University, Aarhus, Denmark; 10 Department of Clinical Immunology, Zealand University Hospital, Køge, Denmark; 11 Department of Clinical Immunology, Odense University Hospital, Odense, Denmark; 12 Department of Research Medicine, University of Southern Denmark, Odense, Denmark; 13 Department of Clinical Immunology, Aalborg University Hospital, Aalborg, Denmark; 14 Department of Infectious Diseases, Aarhus University Hospital, Aarhus, Denmark; 15 University Research Clinic for Cancer Screening, Randers Regional Hospital, Randers, Denmark; 16 Department of Infectious Diseases, Aalborg University Hospital, Aalborg, Denmark; 17 Department of Clinical Medicine, Aalborg University, Odense, Denmark; 18 Department of Infectious Diseases, Odense University Hospital, Odense, Denmark; 19 Department of Infectious Diseases, Zealand University Hospital, Roskilde, Denmark; 20 Department of Science and Environment, University of Roskilde, Roskilde, Denmark; 21 Department of Clinical Research, North Zealand Hospital, Hillerød, Denmark; 22 Department of Public Health, University of Copenhagen, Copenhagen, Denmark; 23 Copenhagen Emergency Medical Services, Copenhagen, Denmark

## Abstract

**Background:**

People experiencing homelessness (PEH) and associated shelter workers may be at higher risk of infection with “Severe acute respiratory syndrome coronavirus 2” (SARS-CoV-2). The aim of this study was to determine the prevalence of SARS-CoV-2 among PEH and shelter workers in Denmark.

**Design and methods:**

In November 2020, we conducted a nationwide cross-sectional seroprevalence study among PEH and shelter workers at 21 recruitment sites in Denmark. The assessment included a point-of-care test for antibodies against SARS-CoV-2, followed by a questionnaire. The seroprevalence was compared to that of geographically matched blood donors considered as a proxy for the background population, tested using a total Ig ELISA assay.

**Results:**

We included 827 participants in the study, of whom 819 provided their SARS-CoV-2 antibody results. Of those, 628 were PEH (median age 50.8 (IQR 40.9–59.1) years, 35.5% female) and 191 were shelter workers (median age 46.6 (IQR 36.1–55.0) years and 74.5% female). The overall seroprevalence was 6.7% and was similar among PEH and shelter workers (6.8% vs 6.3%, *p* = 0.87); and 12.2% among all participants who engaged in sex work. The overall participant seroprevalence was significantly higher than that of the background population (2.9%, *p* < 0.001). When combining all participants who reported sex work or were recruited at designated safe havens, we found a significantly increased risk of seropositivity compared to other participants (OR 2.23, 95%CI 1.06–4.43, *p* = 0.02). Seropositive and seronegative participants reported a similar presence of at least one SARS-CoV-2 associated symptom (49% and 54%, respectively).

**Interpretations:**

The prevalence of SARS-CoV-2 antibodies was more than twice as high among PEH and associated shelter workers, compared to the background population. These results could be taken into consideration when deciding in which phase PEH are eligible for a vaccine, as part of the Danish national SARS-CoV-2 vaccination program rollout.

**Funding:**

TrygFonden and HelseFonden.

**Supplementary Information:**

The online version contains supplementary material available at 10.1186/s12889-022-13642-7.

## Summary of key points

Homeless and shelter workers had a SARS-CoV-2 seroprevalence of 6.7%. Those who reported sex work were at a fourfold elevated risk of being SARS-CoV-2 seropositive. There was no significant association between reported symptoms and seropositivity, nor between substance abuse and seropositivity. 

## Introduction

Severe acute respiratory syndrome coronavirus 2 (SARS-CoV-2), has since its emergence in China in 2019 caused a global pandemic. As of April 26^th^ 2021 an estimated 146 million people worldwide were infected and more than 3 million people has died from SARS-CoV-2 [1]. The first confirmed case of SARS-CoV-2 in Denmark was detected on February 27^th^, 2020. Since then, there have been more than 246 460 confirmed cases of SARS-CoV-2 in Denmark [2]. More than 12 880 of the Danish SARS-CoV-2 patients were hospitalized between January 2020 and March 2021.

Vulnerable groups including people experiencing homelessness (PEH) have challenges in accessing health care systems and public health information [3], including that of SARS-CoV-2. A French study found the overall seroprevalence among PEH to be 52.1% which was 4.3 times higher than the modelled estimate for the general population in Ile de France (12%) [4]. Limited knowledge of protection against SARS-CoV-2 among vulnerable individuals such as PEH is likely to increase the risk of infection for both PEH and people in their proximity, such as shelter workers. Additionally, the recommended guidelines to prevent the spread of SARS-CoV-2 might not be feasible due to inadequate access to handwash, protective equipment and difficulties in practicing social distancing [5, 6]. An estimated 6,431 (0.1%) Danes are categorized as homeless, of whom 2.666 (41.5%) are registered in the Capital Region of Denmark and the rest are distributed throughout the largest cities in Denmark [7, 8]. PEH have more physical and mental health issues than the background population [9, 10] and often engage in substance abuse [7], which could increase their risk of infection and of a serious course of disease by SARS-CoV-2 [11, 12]. Furthermore, a fear of experiencing serious withdrawal symptoms may prevent PEH from both testing and subsequent self-isolation. Furthermore, crowded living conditions in shelters and public spaces where PEH reside, constitute a potential risk of becoming epicenters, as congregate settings have proven to be associated to high exposure to SARS-CoV-2 [4].

Some PEH engage in sex work which may further increase the risk of infection with SARS-CoV-2, due to direct physical contact with clients [13]. In addition, sex workers are known to have a high prevalence of HIV [14] and other underlying health conditions [15, 16], which may add to their risk of SARS-CoV-2 progressing to severe illness [12].

Systematic screening for SARS-CoV-2 antibodies is an important tool in the surveillance of the current pandemic. Information on the prevalence of SARS-CoV-2 infection among vulnerable groups such as PEH is important to assess the need for preventative measures in such groups, to provide information about support estimations of the overall prevalence of SARS-CoV-2 infection and to help guide the public health response in the future. This study is part of the national surveillance study “Testing Denmark”, aimed at assessing the extent and impact of SARS-CoV-2 infection in Denmark [17]. The aim of the present study was to determine SARS-CoV-2 seropositivity among PEH and shelter workers in Denmark, and to study risk factors for infection and clinical presentation in PEH.

## Design and methods

### Study design and sampling strategy

Between November 2^nd^ and 20^th^ 2020 we conducted a nationwide, cross-sectional seroprevalence study to determine the prevalence of SARS-CoV-2 antibodies among PEH. In addition, we also included shelter workers at the recruitment sites. SARS-CoV-2 antibody testing was done using a rapid point-of care SARS-CoV-2 antibody test (POCT) and participants were invited to fill in a questionnaire (supplementary file 1) in collaboration with a trained project employee, at the same time as the test was performed. We recruited participants from 21 sites in the four biggest cities in Denmark; Copenhagen, Aarhus, Aalborg and Odense. The recruitment sites were shelters, supervised sites for intravenous drug abusers, food distribution sites, meeting places and day/night cafés. In the week prior to our visit, written information was distributed by shelter workers at the recruitment sites notifying the participants of our project. To ensure a high attendance and inclusion, recruitment sites were visited several times, on different days and at different time of the day, including weekends and evenings. The objective was to invite all PEH and shelter workers at each of the 21 assessed sites to participate. However, participation was voluntary. Participants were encouraged to wait around for15 minutes for their test results, but in case they did not want to, they were contacted if the test result was positive. Most came back throughout the day to receive their test result.

### Background population

All Danish blood donations are routinely screened for the presence of SARS-CoV-2 antibodies since October 2020. Blood donations take place in all five Danish administrative regions [18] and donors are 17–69 years old. The seroprevalence estimates from the period between the 1^st^ and 22^nd^ of November 2020, are used in this study as proxies for the SARS-CoV-2 infection in the background population.

### Point of care test

SARS-CoV-2 antibodies in PEH and shelter workers were detected in whole blood, by use of the OnSite COVID-19 IgG/IgM Rapid Test (CTK Biotech inc., Poway, California, United States of America) according to the manufacturer’s recommendations. This POCT is a single use lateral flow chromatographic immunoassay rapid test, intended for qualitative detection and differentiation of SARS-CoV-2 immunoglobulin M (IgM) and immunoglobulin G (IgG) antibodies. Blood was extracted from the fingertip. Test results were read after 15 min by a trained project employee. Participants were categorized as seropositive if they were either IgG and/or IgM positive. The test sensitivity and specificity is 96.86% (95% confidence interval (95% CI:93.66%-98.47%) and 99.39% (95% CI 97.80%-99.83%) respectively[19], as reported from the manufacturer. In house validation of CTK’s POCT showed a slightly lower sensitivity of 90% and a specificity of 100% [20].

### Enzyme-Linked Immunosorbent Assay (ELISA)

In contrast to PEH and shelter workers, blood donors were screened for seropositivity using an Enzyme-Linked Immunosorbent Assay (ELISA), with a sensitivity of 96.7% (95% CI 92.4–98.6) and specificity of 99.5% (95% CI 98.7–99.8) (Beijing Wantai Biological Pharmacy Enterprise, China). Participants who were either IgG and/or IgM positive, were categorized as seropositive.

### Questionnaire

Participants were invited to fill in a questionnaire provided at the recruitment site, comprised of questions covering sociodemographic characteristics, physical health, self-reported height and weight, use of drugs and alcohol, sex work, co-morbidities, symptom manifestations and the use of personal protective equipment against SARS-CoV-2. Alcohol abuse was defined as drinking more than the national recommendations of < 1 or < 2 beverages per day for women and men, respectively [21]. The questionnaire was filled out with the assistance of a trained project employee. Personal data was collected using a web-based electronic data capture tool (Research Electronic Data Capture, REDCap) [22, 23].

### Study group

PEH were defined as people living rough, in emergency accommodation, in accommodation for the homeless or living in non-conventional dwellings due to lack of housing, in accordance with the ETHOS (European Typology on Homelessness and Housing Exclusion) classification [24] established by the FEANTSA (European Federation of National Organizations Working with the Homeless) [25]. Sex workers were defined as individuals either having reported to be engaged in sex work in the questionnaire and/or being included at one of the designated safe havens for sex workers, Reden or Reden International [26]. The safe havens are drop-in centers that offer sex workers a break from the streets, providing food, counselling and a free and anonymous health clinic. Shelter workers were defined as people either working or volunteering at the recruitment sites, by providing support to the residents and visitors [27].

### Primary outcome

The primary outcome was the proportion of the study population with a positive antibody test (IgG and/or IgM) for SARS-CoV-2.

### Statistical analysis

The estimated sample size was 491, as we assumed that the risk of being seropositive were two times higher for PEH compared to the estimated 3% seroprevalence in the background population. This was calculated with an α of 0.05 and power of 80%. Seropositivity is presented as numbers (n) and percentage (%) with 95% confidence intervals (95% CI). Baseline characteristics and exposures are presented as n (%) for factors and median and interquartile range (IQR)) for numeric variables as appropriate. The body mass index (BMI) was defined as weight in kilograms divided by height in meters squared. BMI was calculated from self-reported height and weight. We calculated the true seroprevalence for both the POCT and ELISA, by adjusting the apparent (crude) seroprevalence in our study group for the sensitivity and specificity of the POCT and ELISA. The calculations were done using the epiR package and by use of the method suggested by Rogan and Gladen to calculate a true prevalence with a 95% CI adjusted for sensitivity and specificity of a test [28]. Possible associations between exposures and primary outcome were reported as odds ratios (OR) and presented with 95% CI. Answers with “do not know” were classified as missing and answers marked “not relevant” were classified as “no”. We tested for significance using Fischer’s exact test. Significant risk factors of seropositivity among sex workers were combined in a multivariate logistic regression model including region of stay, age and gender. *P*-values < 0.05 were considered significant. Analyses were performed using RStudio version 1.2.5001 [29].

## Results

### Characteristics

**Fig. 1:**
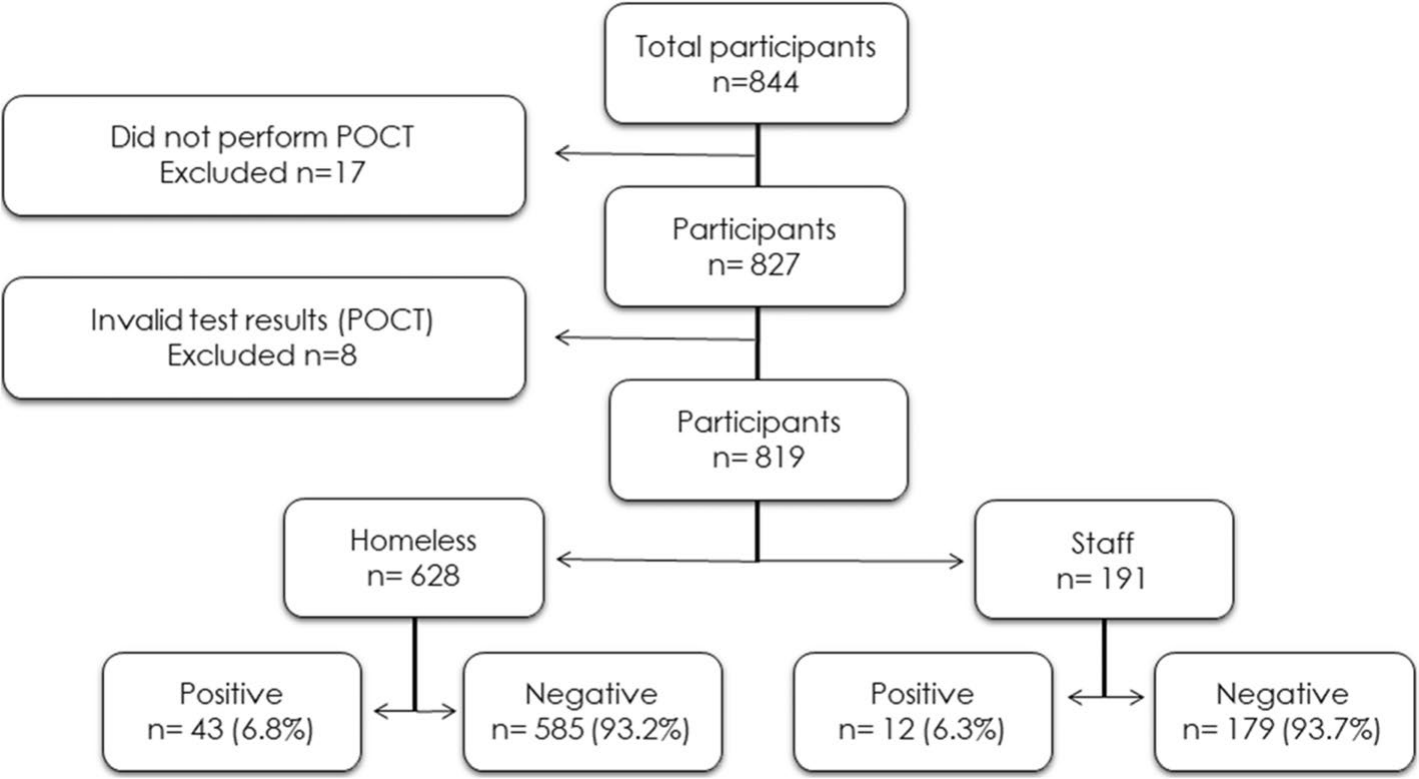
Flow chart reporting the flow of participants through each stage

We recruited a total of 827 participants between the 2^nd^ and 20^th^ of November 2020 of which 819 provided their SARS-CoV-2 antibody result as illustrated in Fig. 1. Participants were recruited from 21 recruitment sites placed in Copenhagen (*n* = 351), Odense (*n* = 128), Aarhus (*n* = 144) and Aalborg (*n* = 144). Fifty-two participants did not register their test location. The recruited participants included 628 PEH (median age 50.8 (IQR 40.9–59.1) years, 35.5% female) and 191 shelter workers (median age 46.6 (IQR 36.1–55.0) years, 74.5% female). Baseline characteristics of the cohort are shown in Table 1, stratified first on PEH and shelter workers and later according to seropositivity. The PEH were older (*p* < 0.001), had a lower BMI (*p* = 0.02), were more likely to be male (*p* < 0.001) and smoke (*p* < 0.001) compared to shelter workers. The only significant difference between the seropositive and seronegative participants, was significantly higher BMI among the seropositive group (*p* < 0.001).

**Table 1:** Baseline characteristics of the study cohort, stratified by shelter workers and people experiencing homelessness (PEH) and by seropositive or seronegative status

	**PEH**	**Shelter Workers**	**p**
**n**	628	191	
**Seropositive (%)**	43 (6.8)	12 (6.3)	0.914
**Age (median [IQR])**	50.83 [40.86, 59.14]	46.62 [36.09, 54.99]	< 0.001
**Gender (%)**			< 0.001
Female	219 (34.9)	140 (73.3)	
Male	396 (63.1)	48 (25.1)	
Other	2 (0.3)	0 (0.0)	
NA	11 (1.8)	3 (1.6)	
**Body mass index (mean (SD))**	24.75 (5.02)	25.92 (5.42)	0.024
**Smoker (%)**	498 (79.3)	89 (46.6)	< 0.001
**Alcohol use (%)**			< 0.001
Yes	307 (48.9)	166 (86.9)	
NA	32 (5.1)	5 (2.6)	
**Previously tested (%)**			< 0.001
Yes	371 (59.1)	154 (80.6)	
NA	22 (3.5)	3 (1.6)	
	**Seronegative**	**Seropositive**	**p**
**n**	764	55	
**PEH (%)**	585 (76.6)	43 (78.2)	0.914
**Age (median [IQR])**	49.09 [38.89, 58.15]	53.23 [45.90, 57.88]	0.093
**Gender (%)**			0.650
Female	332 (43.5)	27 (49.1)	
Male	416 (54.5)	28 (50.9)	
Other	2 (0.3)	0 (0.0)	
NA	14 (1.8)	0 (0.0)	
**Body mass index (mean (SD))**	24.79 (4.94)	27.66 (6.66)	< 0.001
**Smoker (%)**	554 (72.5)	33 (60.0)	0.067
**Alcohol use (%)**			0.048
Yes	441 (57.7)	32 (58.2)	
NA	31 (4.1)	6 (10.9)	
**Previously tested (%)**			0.553
Yes	490 (64.1)	35 (63.6)	
NA	22 (2.9)	3 (5.5)	

PEH: people experiencing homelessness. Seropositive: SARS-CoV-2 IgM and/or IgG antibodies detected in POCT. Smoker: either previously or currently smoking. Alcohol use: intake of alcohol within the past 12 months. Previous SARS-CoV-2 Tested: have previously been tested for SARS-CoV-2. IV drugs: use of intravenous drugs. Smoked drugs: drugs that were reported smoked. Alcohol use: intake of alcohol within the past 12 months. Never COVID-19 Tested: never previously been tested for COVID-19

Data are *n* (%) unless otherwise specified. P values are calculated using Fisher’s exact test

### Seroprevalence

Of the 819 participants, 55 (6.7%) were seropositive. We found that 43 of 628 (6.8%) PEH and 12 of 191 (6.3%) shelter workers were seropositive, the prevalence in the two groups was not significantly different (*p* = 0.87).

### Seroprevalence compared to the background population

**Fig. 2:**
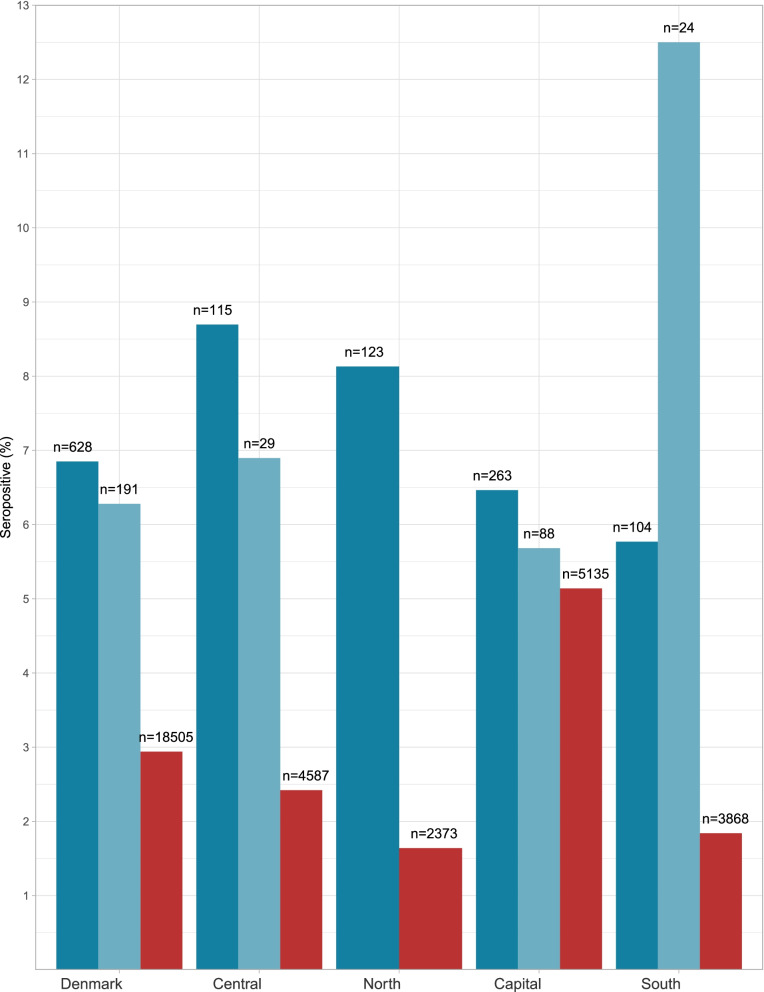
Seroprevalence among PEH and shelter workers, compared to the background population

In the period between the 1^st^ and 22^nd^ of November 2020, the background population (*n* = 18,505) had a 2.9% seroprevalence. This group was characterized by a median age of 43 (IQR 29–54) years and a higher proportion (47.9%) were women. Taken together, the participants in our study (PEH and shelter workers combined) were at a significantly higher risk of seropositivity than the background population (OR 2.37, 95%CI 1.75–3.17, *p* < 0.001). Figure 2 illustrates the regional SARS-CoV-2 seroprevalence in PEH, shelter workers and the background population. The seroprevalence among PEH alone was also significantly higher than that in the background population (OR 2.43, 95%CI 1.72–3.35, *p* < 0.001). The subset of shelter workers (*n* = 191) in general was significantly associated with seropositivity compared to the background population (OR 2.21, 95%CI 1.12–4.00). In an adjusted analysis for test characteristics of the POCT we found an estimated true seroprevalence of 7.46% (95% CI 5.77–9.60). The estimated true seroprevalence as measured by ELISA was 2.50% (95%CI 2.26–2.76).

### Risk factors for attracting infection

Table 2 shows the prevalence of risk factors (drugs, alcohol, sex work) in the study population, stratified by seropositivity. Tobacco was not considered a drug. None of the lifestyle risk factors were significantly associated to seropositivity. A total of 285 (45.4%) PEH reported use of drugs, the most commonly used were cocaine (18.6%) and heroin (15.6%). Among the seropositive PEH, heroin (14.5%) was the most common drug while the seronegative PEH were more likely to use cocaine (16.9%). Further, we found that 326 (51.9%) PEH were smokers; of those 326 (51.9%) smoked tobacco and 180 (28.7%) smoked cannabinoids. Other reported smoked substances were cocaine (3.5%) and heroin (2.5%). A total of 307 (51.5%) PEH reported some use of alcohol within the past year. Of the 191 shelter workers only 3 (1.6%) reported using drugs, all of which were of cannabinoids. Further, 166 (89.2%) reported any use of alcohol within the past year. PEH were significantly more likely to abuse alcohol (*p* < 0.001) and use drugs (*p* < 0.001), compared to the shelter staff.

**Table 2:** Characteristics and risk factors stratified according to seropositivity stratified by PEH and shelter worker (IgM and/or IgG)

	**Seropositive PEH**	**Seronegative PEH**	**p**
**n**	43	585	
**Drug abuse (%)**	9 (20.9)	221 (37.8)	0.040
IV drugs (%)	8 (18.6)	154 (26.3)	0.349
Smoked drugs (%)	11 (25.6)	192 (32.8)	0.418
**Alcohol use (%)**			0.019
Yes	21 (48.8)	286 (48.9)	
NA	6 (14.0)	26 (4.4)	
**Alcohol abuse (%)**			0.549
Yes	8 (18.6)	132 (22.6)	
NA	28 (65.1)	331 (56.6)	
**Sex work (%)**	8 (18.6)	64 (10.9)	0.202
**Contact with known SARS-CoV-2 infected person:**			
**15 min Contact (%)**			0.550
Yes	4 (9.3)	45 (7.7)	
NA	37 (86.0)	527 (90.1)	
**Physical Contact (%)**			0.377
yes	2 (4.7)	14 (2.4)	
NA	36 (83.7)	528 (90.3)	
**Social Circle (%)**			0.443
Yes	8 (18.6)	70 (12.0)	
NA	7 (16.3)	100 (17.1)	
	**Seropositive Shelter Workers**	**Seronegative Shelter Workers**	**p**
**n**	12	179	
**Drug abuse (%)**	0 (0.0)	3 (1.7)	1.000
IV drugs (%)	0 (0.0)	0 (0.0)	
Smoked drugs (%)	0 (0.0)	3 (1.7)	1.000
**Alcohol use (%)**			0.809
Yes	11 (91.7)	155 (86.6)	
NA	0 (0.0)	5 (2.8)	
**Alcohol abuse (%)**			0.381
Yes	2 (16.7)	14 (7.8)	
NA	1 (8.3)	37 (20.7)	
**Sex work (%)**	1 (8.3)	1 (0.6)	0.273
**Contact with known SARS-CoV-2 infected person:**			
**15 min Contact (%)**			0.640
Yes	1 (8.3)	27 (15.1)	
NA	11 (91.7)	146 (81.6)	
**Physical Contact (%)**			0.451
Yes	1 (8.3)	12 (6.7)	
NA	11 (91.7)	146 (81.6)	
**Social Circle (%)**			0.423
Yes	2 (16.7)	60 (33.5)	
NA	4 (33.3)	39 (21.8)	

PEH: people experiencing homelessness. IV drugs: use of intravenous drugs. Smoked drugs: drugs that were reported smoked. Alcohol use: intake of alcohol within the past 12 months. Alcohol Abuse: drinking more than the national recommendations of 2 drinks or less in a day for men or 1 drink or less in a day for women. 15 min. contact: having been in the same room as someone with an ongoing SARS-CoV-2 infection for 15 min or longer. Physical contact: having had close bodily contact with a SARS-CoV-2 positive individual. Social circle: aware of anyone in close contacts who had SARS-CoV-2. Data are *n* (%) unless otherwise specified. P values are calculated using Fisher’s exact test

### Sex work

Of the 72 (11.5%) PEH who engaged in sex work, 8 (11.1%) were seropositive. Table 3 illustrates characteristics and risk factors of PEH stratified by sex work. Sex workers were younger, more likely to be female and less likely to report smoking and/or IV drug use than other PEH (all *p* < 0.001). Further, two (1.0%) shelter workers reported having engaged in sex work, of whom one was found to be SARS-CoV-2 seropositive. The seroprevalence among all participants, both PEH and shelter workers, engaging in sex work (*n* = 74) was 12.2%. Sex workers were 1.9 times more likely to be seropositive compared to non sex workers (OR 1.9). For all participants engaging in sex work (*n* = 74), there was a significantly increased risk of seropositivity for IgG antibodies compared to the rest of the study group (OR 3.00, 95%CI 1.06–7.49, *p* = 0.02). However, for the combined seropositivity (IgG and/or IgM antibodies) the difference did not reach significance (OR 2.10, 95%CI 0.87–4.60, *p* = 0.08).

**Table 3:** Characteristics and risk factors for people experiencing homelessness (PEH), stratified by engagement in sex work

	Sex Workers	PEH not engaging in sex work	p
**n**	72	556	
**Seropositive (%)**	8 (11.1)	35 (6.3)	0.202
**Age (median [IQR])**	41.43 [32.51, 49.96]	51.57 [41.64, 59.52]	< 0.001
**Gender (%)**			< 0.001
Female	68 (94.4)	151 (27.2)	
Male	3 (4.2)	393 (70.7)	
Other	1 (1.4)	1 (0.2)	
NA	0 (0.0)	11 (2.0)	
**Smoker (%)**	44 (61.1)	454 (81.7)	< 0.001
**Drug abuse (%)**	17 (23.6)	213 (38.3)	0.021
IV Drugs (%)	6 (8.3)	156 (28.1)	0.001
Smoked drugs (%)	19 (26.4)	184 (33.1)	0.312
**Alcohol use (%)**			
Yes	27 (37.5)	280 (50.4)	< 0.001
NA	16 (22.2)	16 (2.9)	
**Alcohol abuse (%)**			
Yes	10 (13.9)	130 (23.4)	0.172
NA	47 (65.3)	312 (56.1)	
**Contact with known SARS-CoV-2 infected person:**			
**15 Min Contact (%)**			
Yes	3 (4.2)	46 (8.3)	0.467
NA	67 (93.1)	497 (89.4)	
**Body Contact (%)**			
Yes	1 (1.4)	15 (2.7)	0.610
NA	67 (93.1)	497 (89.4)	
**Social Circle (%)**			
Yes	8 (11.1)	70 (12.6)	0.036
NA	20 (27.8)	87 (15.6)	
**Previously tested (%)**			
Yes	39 (54.2)	332 (59.7)	< 0.001
NA	15 (20.8)	7 (1.3)	

Sex workers: PEH engaging in sex work. PEH: People experiencing homelessness. Seropositive: SARS-CoV-2 IgM and/or IgG antibodies detected in POCT. Smoker: either previously or currently smoking. IV drugs: use of intravenous drugs. Smoked drugs: drugs that were reported smoked. Alcohol use: intake of alcohol within the past 12 months. Alcohol Abuse: drinking more than the national recommendations of 2 drinks or less in a day for men or 1 drink or less in a day for women. Never COVID-19 Tested: never previously been tested for COVID-19. Data are *n* (%) unless otherwise specified. P values are calculated using Fisher’s exact test

We included a total of 33 shelter workers working at designated safe havens for sex workers, of whom 4 (12.1%) were found to be seropositive. Shelter workers at designated safe havens for sex workers had a far greater infection rate than other shelter workers, however, the difference was not significant (OR 2.57, 95%CI 0.53–10.38, *p* = 0.13). Further, there was no difference in seropositivity between shelter workers at designated safe havens (12.1%) and all sex workers (12.2%) (OR 1.38, 95%CI 0.32–5.26, *p* = 0.752). When combining all participants who reported sex work or were recruited at designated safe havens, shelter workers and sex workers (*n* = 107), we found a significantly increased risk of seropositivity (IgG and/or IgM antibodies) compared to other participants (OR 2.23, 95%CI 1.06–4.43, *p* = 0.02). In a multivariate logistic regression model of region of stay (supplementary table 1), being a sex worker or working at a designated safe haven remained a significant risk factor of seropositivity compared to PEH who does not engage in sex work (OR 2.4, 95% CI 1.12–4.70, *p* = 0.02).

### Symptoms and self-reported illness

Of the 628 PEH, 57 (9.1%) suspected previous infection with SARS-CoV-2 of whom 8 (14%) were seropositive. A total of 371 (59.1%) PEH reported being previous tested for SARS-CoV-2, of whom 11 (3.0%) were reportedly positive at the time. However, of those only 3 (27.3%) tested positive for SARS-CoV-2 antibodies in our study. Of the shelter workers, 154 (80.6%) reported previous testing for SARS-CoV-2. No shelter workers reported having previously tested positive for SARS-CoV-2, but 21 (11%) suspected previous infection with SARS-CoV-2 of whom 5 (23.8%) were seropositive.

Seventeen of 43 (39.5%) seropositive PEH and 10 of the 12 (83.3%) seropositive shelter workers reported having had symptoms. Overall, a total of 441 (53.8%) participants in our study group reported having had one or more symptoms. Of those, 15 (3.3%) reported being hospitalized at the time of their symptoms. Among the PEH 303 (48.2%) reported experiencing one or more symptom, of whom 26 (8.6%) though it was attributable to SARS-CoV-2. Of the shelter workers 138 (72.3%) reported one or more symptom, and of those 20 (14.5%) thought the symptoms were attributable to SARS-CoV-2; significantly more than among the PEH (OR 2.79, 95%CI 1.94–4.06, *p* < 0.001). Similarly, shelter workers were more likely than PEH to have more than three symptoms (OR 2.11, 95%CI 1.47–3.03, *p* < 0.001). The most common symptoms reported in the combined cohort were fever (23.2%) and shivers (18.5%). However, there was no observed significant association between experiencing symptoms and seropositivity (OR 0.82, 95%CI 0.45–1.47, *p* = 0.49). Table 4 shows symptoms stratified by seropositivity. Symptoms in PEH and shelter workers is illustrated in supplementary table 2.

**Table 4:** Symptoms reported by the participants, stratified by serology findings

	Seropositive	Seronegative	p
**n**	55	764	
Any symptom (%)	27 (49.1)	414 (54.2)	0.554
Fever ≥ 38 °C (%)	8 (14.5)	182 (23.8)	0.159
Chills (%)	10 (18.2)	142 (18.6)	1.000
Loss of Smell (%)	8 (14.5)	63 (8.2)	0.175
Loss of Taste (%)	7 (12.7)	54 (7.1)	0.201
Sore Throat (%)	12 (21.8)	238 (31.2)	0.194
Cough (%)	16 (29.1)	315 (41.2)	0.103
Shortness of breath (%)	8 (14.5)	154 (20.2)	0.404
≥ 3 Symptoms (%)	11 (20.0)	204 (26.7)	0.351

Symptoms experienced since March 1^st^, 2020. ≥ 3 Symptoms; participants who registered three or more symptoms

Data are *n* (%) unless otherwise specified. P values are calculated using Fisher’s exact test

### Use of protective means against SARS-CoV-2 infection

**Fig. 3:**
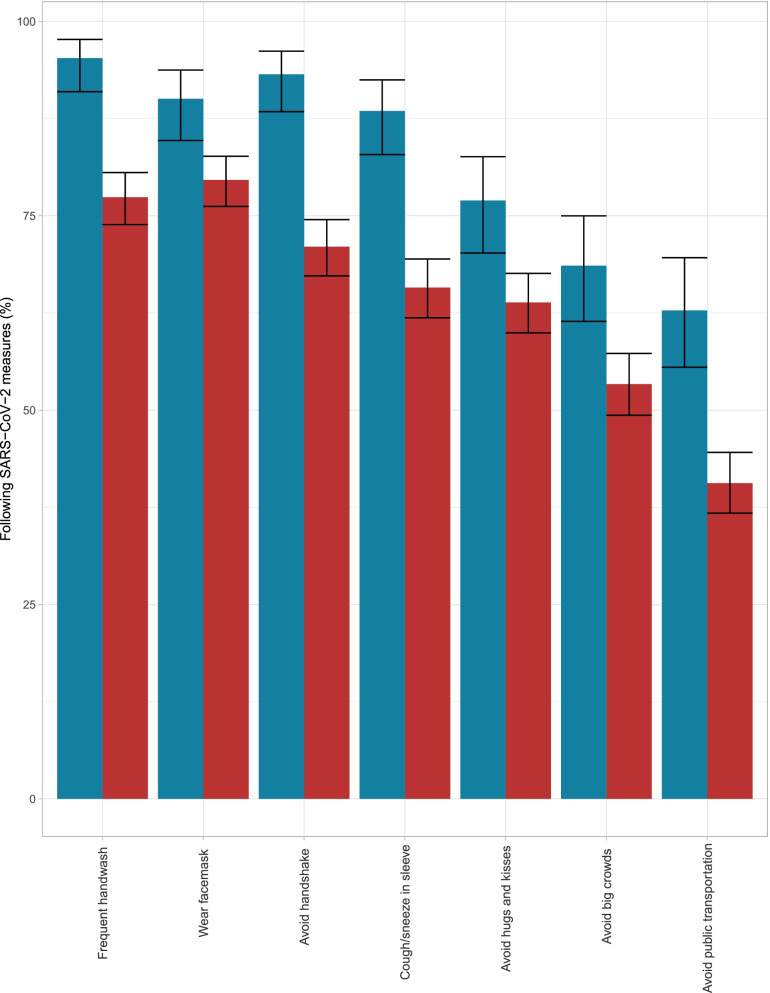
Percentage of PEH and shelter workers who follow the national SARS-CoV-2 measures and guidelines

Figure 3 shows use of protective means against SARS-CoV-2 in PEH compared to shelter workers. Only 25 (4%) of the PEH reported that they did not follow any of the recommended guidelines. Among the shelter workers only 3 (1.6%) reported not following the guidelines. The supplementary Fig. 1 illustrates use of protective means against SARS-CoV-2 in seropositive and seronegative participants. There was no significant association between following any given guideline and seropositivity.

## Discussion

To our knowledge, our study is the first to investigate and evaluate the nationwide prevalence of SARS-CoV-2 infection among PEH. We found that the seroprevalence among PEH was twice that of the background population. Furthermore, sex workers and shelter workers at designated safe havens were at increased risk of infection with SARS-CoV-2, independent of region. We found that seropositive PEH were less likely to report symptoms, compared to seropositive shelter workers. The results further suggest that almost all PEH follow one or more national SARS-CoV-2 prevention measure, as illustrated in Fig. 3. The high seroprevalence among PEH found in our study suggest that PEH are at elevated risk of infection with SARS-CoV-2 which could be taken into consideration when deciding in which phase they are eligible for a vaccine, as part of the national SARS-CoV-2 vaccination program rollout. Our findings are consistent with findings in previous studies[4, 30–32], with elevated prevalence of SARS-CoV-2 for people living in precarious conditions, relative to the background population. This is consistent with living in overcrowded conditions, a main risk factor associated with infection of SARS-CoV-2. The observed increased seroprevalence in sex workers compared to those who did not engage in sex work, is in accordance with the recent statement from UNAIDS (the joint United Nations Programme on HIV/AIDS) which emphasized how sex workers are risking their health by working during the current SARS-CoV-2 pandemic in order to provide for themselves [33]. Further, we believe that national SARS-CoV-2 measures such as social distancing are simply not feasible for sex workers as their work requires some level of close physical contact with clients and self-isolation could result in a total loss of income. These results show that the risk of contracting SARS-CoV-2 should be added to the risks experienced by sex workers.

In previous studies around half the seropositive participants are reporting symptoms attributable to SARS-CoV-2 [34–36]. Symptom prevalence in our study is consistent with previous findings on PEH and implies a high proportion of asymptomatic infections [4, 30, 37, 38]. Additional explanations might include difficulties in recalling previous symptoms and differentiating symptoms attributable to substance abuse and SARS-CoV-2. Thus, symptom assessment in PEH might not be predictive for SARS-CoV-2. The high prevalence of substance abuse among PEH is consistent with previous national findings on PEH [7]. Recent studies suggest that suffering from a substance use disorder increases the risk of contracting SARS-CoV-2, while also facing a worse outcome than the background population [3, 39]. We found that seropositive participants had a higher BMI compared to seronegative participants. The seropositive participants in our study were generally overweight [40], with a mean BMI of 27.7. The Danish Health Authority has previously declared individuals who are overweight at increased risk of developing severe illness from SARS-CoV-2. They identify multiple possible reasons as to why overweight individuals are at increased risk, counting impaired immune function, comorbidities and low physical activity levels [11].

### Strengths and Limitations

Our study has several limitations. First, the cross-sectional study designs do not allow determination of time of infection nor provide information on when participants became seropositive. Individuals who might already have tested positive by PCR at an earlier point in time, might have chosen not to participate in this study. Individuals anticipating a positive result might have chosen not to participate, fearful of the consequences such as isolation. If so, our results could be biased and the seroprevalence be underestimated. However, our impression is that the desire to participate and get tested was high (9.8% of an estimated 6 431 homeless people in Denmark). Of the 827 participants in our study group, 544 (67.0%) reported having previously been tested (nasopharyngeal swab and/or antibody test) and still participated. Further, questions on sociodemographic characteristics, physical health, use of drugs and alcohol, co-morbidities, symptom manifestations and the use of personal protective equipment against SARS-CoV-2 were self-reported, therefore an information bias could have affected our results.

We compared our seropositivity findings to that of blood donors serving as a proxy to the background population, with some limitations to consider. First, blood donors are required to have a good general health and are ineligible to donate blood if they have ever engaged in sex work, have certain medical conditions, travel to certain international destinations and/or receive certain immunizations. Seropositivity could as a result potentially be lower among blood donors compared to the background population. However, due to their good health, blood donors are more represented in the labor market and so more at risk of SARS-CoV-2 infection.

Another limitation is that blood donors and our study group were tested using different methods, POCT vs ELISA however both with high and similar sensitivity and specificity. Serology testing with a POCT was chosen for this study as it only requires capillary blood obtained by a finger prick, can be performed as a self-test and provides quick results. We expected that testing by POCT would increase the number of participants, due to the above-mentioned circumstances. Another strength of the POCT is that by not requiring a venous blood sample nor laboratory equipment, it is less costly than a serology test performed in the laboratory and provide more rapid results. Furthermore, communicating test results obtained from an ELISA test would not be possible in the examined group of PEH.

A limitation to the serology approach of this study versus test by PCR, is that the measured antibodies generated in response to SARS-CoV-2 exposure are generated in the weeks after the acute phase of the infection. Recent studies have shown that SARS-CoV-2 IgM antibodies reach a detection threshold 5–7 days after symptom onset [25]. Thus, participants currently or recently infected with SARS-CoV-2 might not have been identified in this study. A strength of serology approach versus test by PCR is that it allows us to detect SARS-CoV-2 antibodies in those categorized as asymptomatic carriers and those with suspected SARS-CoV-2 despite negative PCR results. Our study sought to investigate the seroprevalence among all PEH and risk factors of being seropositive. We did not have the sample size to look at risk factors in subgroups, such as sex workers. Future studies should investigate specific risk factors among these at-risk groups.

## Conclusions

In this study we found a high SARS-CoV-2 seroprevalence among PEH and shelter workers, compared to the background population. There was no significant association between reported symptoms and seropositivity, nor between substance abuse and seropositivity. The results from this study could be taken into consideration when deciding in which phase PEH are eligible for a vaccine, as part of the national SARS-CoV-2 vaccination program rollout. Furthermore, our findings could be of great value in case of future pandemics.

## Supplementary Information

**Additional file 1.**

**Additional file** **2.**

## Data Availability

The datasets used and/or analyzed during the current study are available from corresponding author on reasonable request.
