# Supplementary material for: SARS-CoV-2 antibody prevalence among homeless people and shelter workers in Denmark: a nationwide cross-sectional study
Source: BMC Public Health. 2022 Jun 27;22:1261. doi: 10.1186/s12889-022-13642-7 (PMC9238223; doi:10.1186/s12889-022-13642-7)
Supplement: Supplementary file 1 — Additional file 1. [file 12889_2022_13642_MOESM1_ESM.docx]

**Questionnaire**

| **Introductory questions** | |
| --- | --- |
| What is your date of birth? | __________________________________ |
| What is your gender? | - Man - Woman - Other - Do not wish to answer |
| At what site were you tested? | - Døgnvarmestuen – Aarhus - Værestedet - Aarhus - E-huset - København - Reden - Aarhus - Mændenes hjem - København - Reden - København - Sundholm - København - Mariatjenesten - København - Kompasset - København - Hillerødgade - København - Reden International - København - Café Klare - København - Reden - Odense - Varmestue Østergade - Odense - Varmestue Nørregade - Odense - Redernes Krisecenter - Odense - Kirkens Korshær Varmestue - Odense (Østergade) - Reden - Aalborg - Svenstrupgård - Aalborg - Kirkens Korshær – Aalborg |
| **Questions about your antibody tests**  **This part of the questionnaire concerns the antibody tests you have done in connection with ”Testing Denmark”** | |
| Do you wish to do a finger prick test for COVID-19? | - Yes - No - Do not know |
| What is the result of your finger prick test? | - Negative - Positive for IgM - Positive for IgG - Positive for both IgM and IgG - Inconclusive - I have not done the test/the test failed |
| **Questions about COVID-19 infection**  **This part of the questionnaire concerns whether you have been infected with COVID-19** | |
| If you were to give your best assessment, do you think/know that you have ever been infected with COVID-19? | - Yes, I know/think that I have had COVID-19 - No, I know that I haven’t had/I don’t think that I have had COVID-19 |
| Have you previously been tested for COVID-19? | - Yes - No |
| What type of COVID-19 test have you taken?  Select one or both answer options: | - Swab (sample from throat, nose or pharynx) - Antibody test (finger prick test or blood test) - Don’t know/don’t wish to answer |
| Was one or more of your tests positive? | - Yes - No |
| **Symptoms**  **The next questions concern your general health and whether you have had symptoms of COVID-19. Have you had one or more of the following symptoms since 1 February?  (Select an answer for each option)** | |
| 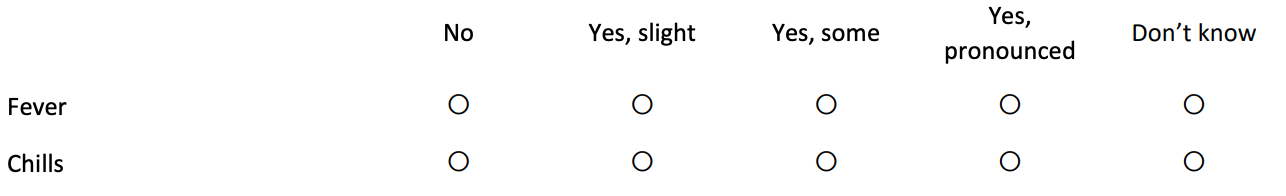  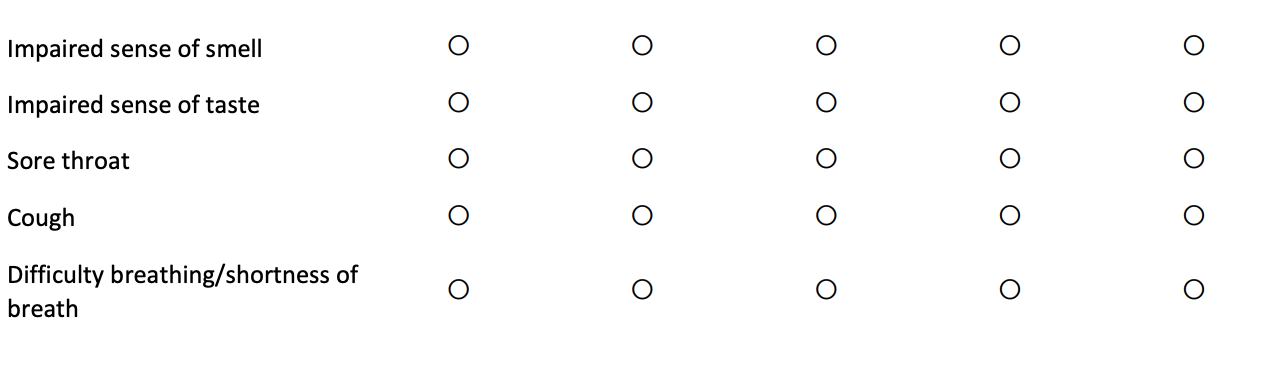 | |
| What do you think caused the symptoms? | - COVID-19 - Other - Do not know |
| Which of the following conditions best describes how you felt when you were feeling the worst, while you had/suspected you had COVID-19? | - I had no symptoms - I had symptoms - I was admitted to hospital - I was admitted and on a ventilator |
| **Risk of COVID-19**  **The following questions concern how great the risk is that you have been exposed to coronavirus.** | |
| 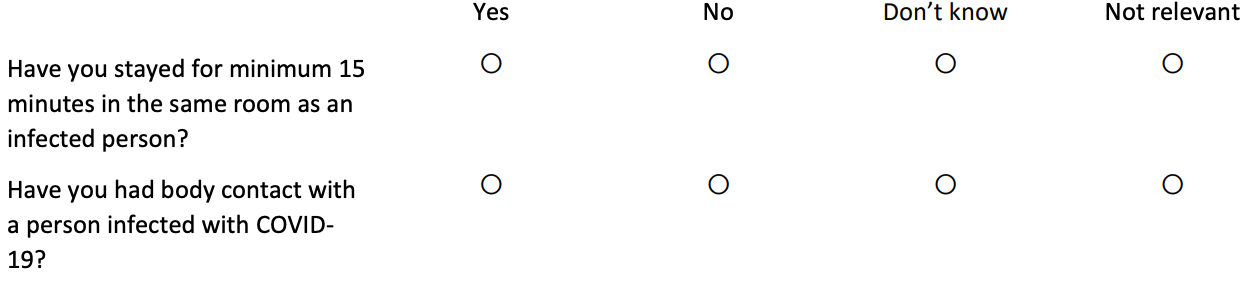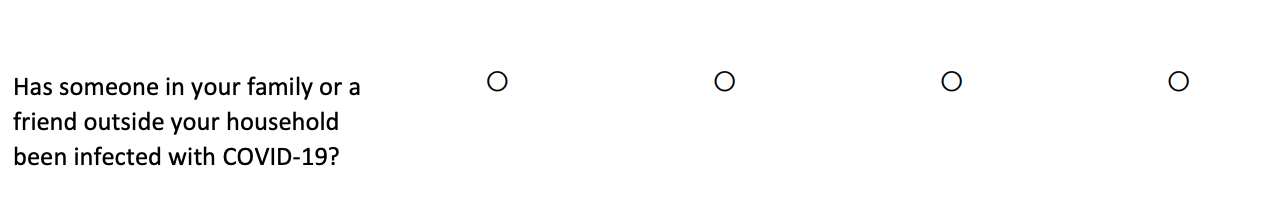  Have anyone in your social circle been infected with COVID-19? | |
| **This part concerns behaviour** | |
| Have you taken any of the following measures due to the risk of COVID-19 infection?  Select all relevant answers: | - I wash hands more often - I cough or sneeze into my sleeve - I wear disposable face masks - I avoid shaking hands - I avoid greeting persons by hugging and/or kissing them on both cheeks - I limit my use of public transport - I avoid places where many people are gathered - None of the above |
| Do you smoke? | - Yes - No - Previously - Do not wish to answer |
| What do you smoke? | _______________________________ |
| How much (on average) do you smoke a day? | _______________________________ |
| On how many days a week do you drink alcohol? | - 0 days - 1-3 days - 4-7 days |
| How many units do you typically drink per week?  *1 unit = 1 regular beer or 1 glass of red/white wine or 1 alcoholic soft drink or 1 alcoholic cider or 1 glass of fortified wine or 1 drink/cocktail or 1 acquavit/shot* | _______________________________ |
| Do you use euphoriant drugs? | - Yes - No - Do not wish to answer |
| If you use euphoriant drugs, which euphoriant drugs do you use? | _______________________________ |
| Do you currently sell sex, or have you sold sex within the last year? | - Yes - No - Do not wish to answer |
| How much do you weigh in kilograms (kg)? | _______________________________ |
| How tall are you in centimetres (cm)? | _______________________________ |
| Did you get an influenza vaccine last autumn/winter 2019?  Select one of the following answer options: | - Yes - No - I do not know/do not remember |
| Did you get an influenza vaccine this autumn/winter 2020? Select one of the following answer options: | - Yes - No - I do not know/do not remember |
