# Supplementary material for: SARS-CoV-2 antibody prevalence among homeless people and shelter workers in Denmark: a nationwide cross-sectional study
Source: BMC Public Health. 2022 Jun 27;22:1261. doi: 10.1186/s12889-022-13642-7 (PMC9238223; doi:10.1186/s12889-022-13642-7)
Supplement: Supplementary file 2 — Additional file 2. [file 12889_2022_13642_MOESM2_ESM.docx]

**Supplementary table 1**: Multivariate logistic regression on significant risk factors of seropositivity.

|  | **OR** | **2.5%** | **97.5%** | **Z value Pr (>\|Z\|)** |
| --- | --- | --- | --- | --- |
| (Intercept) | 0.03 | 0.01 | 1.000000e-01 | 8.71e-07 *** |
| Region Missing | 0.46 | 0.07 | 1.840000e+00 | 0.3287 |
| The Capital Region of Denmark | 0.84 | 0.40 | 1.870000e+00 | 0.6614 |
| The North Denmark Region | 0.80 | 0.32 | 2.000000e+00 | 0.6350 |
| The Region of Southern Denmark | 0.60 | 0.21 | 1.620000e+00 | 0.3211 |
| PEH and shelter workers | 0.91 | 0.43 | 1.820000e+00 | 0.8055 |
| Age | 1.02 | 1.00 | 1.050000e+00 | 0.0442 * |
| Gender, Male | 0.97 | 0.51 | 1.880000e+00 | 0.9254 |
| Gender, Other | 0.00 | NA | 2.754142e+36 | 0.9844 |
| Sex worker, Yes | 2.76 | 1.14 | 6.420000e+00 | 0.0201 * |

**Supplementary table 2**: Symptoms stratified according to PEH and shelter workers.

|  | PEH | Shelter Worker | p |
| --- | --- | --- | --- |
| **n** | 628 | 191 |  |
| **Seropositive (%)** | 43 (6.8) | 12 (6.3) | 0.914 |
| **Any symptom (%)** | 303 (48.2) | 138 (72.3) | <0.001 |
| **Fever ≥38°C (%)** | 116 (18.5) | 74 (38.7) | <0.001 |
| **Chills (%)** | 104 (16.6) | 48 (25.1) | 0.010 |
| **Loss of Smell (%)** | 50 (8.0) | 21 (11.0) | 0.247 |
| **Loss of Taste (%)** | 46 (7.3) | 15 (7.9) | 0.931 |
| **Sore Throat (%)** | 145 (23.1) | 105 (55.0) | <0.001 |
| **Cough (%)** | 229 (36.5) | 102 (53.4) | <0.001 |
| **Shortness of breath (%)** | 127 (20.2) | 35 (18.3) | 0.636 |
| **≥3 Symptoms (%)** | 142 (22.6) | 73 (38.2) | <0.001 |

Symptoms experienced since March 1^st^ 2020. ≥3 Symptoms; participants who registered three or more symptoms.

**Supplementary figure 1:** Percentage of seropositive and seronegative participants, who follow the national SARS-CoV-2 measures and guidelines.


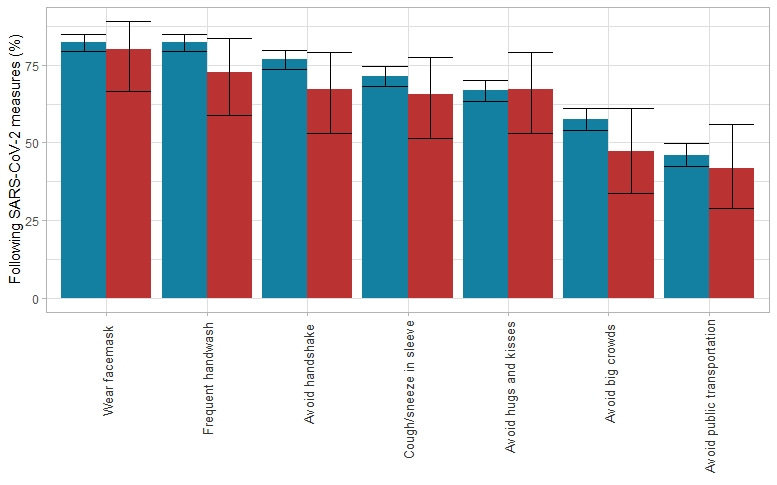


Blue: Seronegative participants; Red: Seropositive participants. The 95% CI of the proportion is illustrated.
